# Improving Completion Rates of Treatment Escalation Plan (TEP) in a London Teaching Hospital: A Quality Improvement Study

**DOI:** 10.7759/cureus.49434

**Published:** 2023-11-26

**Authors:** Saphalya Pattnaik, Ahmadreza Zarifian, Gur Aziz Singh Sidhu, Shahid Punwar

**Affiliations:** 1 Trauma and Orthopaedics, University Hospital Lewisham, London, GBR; 2 Trauma and Orthopaedics, Derby and Burton, Birmmingham, GBR

**Keywords:** dnacpr, pdsa cycles, reducing the cost in orthopedic surgery, quality improvement study, treatment escalation plans

## Abstract

Background

Treatment escalation plans (TEPs) provide enhanced clarity in planning appropriate decision-making in the management of deteriorating patients by explicitly defining a limit of care. These decisions are discussed with patients or their relatives and mutually agreed upon. We aimed to improve staff adherence to the completion of TEPs upon the admission of patients to the orthopedics wards in a London teaching hospital.

Methods

This study employed the Plan-Do-Study-Act (PDSA) methodology to investigate the efficacy of interventions implemented within a hospital setting for adult inpatients receiving orthopedic treatment. The approach adopted was cross-sectional, where a comprehensive audit was conducted on all adult inpatients admitted to the hospital. The initial cycle of the study was conducted in March 2022, followed by the implementation of interventions in the form of an internal algorithm. Subsequently, the second cycle of the study was conducted in November 2022.

Results

We sampled a total of 50 patients (PDSA 1, n=27; PDSA 2, n=23). Following the implementation of a designated local TEP pathway, the proportion of patients with incomplete TEPs fell from 30.4% (n=7, PDSA Cycle 1) to 11.76% (n=2, PDSA Cycle 2).

Conclusions

The study has demonstrated that interventions such as institutional algorithms and departmental meetings can be useful in improving the adherence of staff to complete TEPs. Ongoing training and education can help overcome some of the barriers to TEP completion.

## Introduction

The implementation of a treatment escalation plan (TEP) enables healthcare professionals to engage in proactive discussions with patients and document their preferences regarding various aspects of emergency care and treatment, including cardiopulmonary resuscitation (CPR). This study was conducted within the Lewisham and Greenwich National Health Service (NHS) trust, encompassing two primary locations: the Queen Elizabeth Hospital (QEH) and the University Hospital Lewisham (UHL), which collectively serve a substantial area in southeast London [1].

In July 2018, the trust introduced a policy concerning the treatment escalation plan (TEP), along with corresponding TEP forms for completion. This policy specifically pertains to patients aged 16 years and older who are admitted to the trust as emergency or unplanned cases while excluding those admitted for elective procedures, such as surgical interventions, as well as day case surgery patients and maternity admissions. Following a review conducted by the trust (do not attempt to cardiopulmonary resuscitation) DNACPR task and Finish group in December 2020, the policy underwent updates [1].

The TEP policy outlines the requirement for periodic assessment of the quality of TEP form content and the frequency of completion on an annual basis. In October 2021, a comprehensive cross-divisional audit was conducted on 26,422 adult inpatient emergency admissions during April-September 2021. The findings indicated that upon admission, only 48% of the audited patients had a completed TEP, with the completion rate being lower in UHL (40% vs. 55%). By 48 hours post-admission, 40% of adult inpatients had a TEP completed, with the rate being only 29% at the UHL site. When looking exclusively at the orthopedics department, the completion rates were even lower, with only 28% at UHL and 41% at QEH [1].

According to previous studies [2,3], TEP proformas have been effectively adopted across multiple NHS hospitals. In a prior quality improvement study, the highest utilization rates for TEPs were observed on orthopedic wards for elderly patients, emphasizing the necessity of TEPs for more vulnerable patients [4]. Another study revealed that 96% of patients and families polled thought TEPs would be useful [5]. A further study found that DNACPR forms were insufficient for guiding clinical decisions on therapy escalation and that TEP proformas help not only patients but also treating physicians, particularly on-call junior doctors [6,7].

A retrospective review of 45 cases demonstrated that formal TEP proformas were required to construct visible and unambiguous treatment plans for deteriorating patients, especially given the complexities of their care [8]. Another study demonstrated that the use of standardized TEP proformas considerably increased the clarity of recorded treatment choices [9], while yet another study discovered that on-call clinicians regarded documented treatment ceilings as extremely beneficial [10].

In response to the COVID-19 pandemic, the National Institute for Health and Care Excellence (NICE) rapidly recommended discussing the ceiling of care decisions with patients and families in a timely manner and documenting these decisions through a TEP [11].

## Materials and methods

Settings

This study utilized a plan-do-study-act (PDSA) approach and a cross-sectional methodology to evaluate the completeness of TEPs provided for adult inpatients admitted for orthopedic surgery at the hospital. The study was approved by the hospital’s clinical governance department (Number 7214) and deemed exempt from ethical review due to the nature of this study.

Inclusion and exclusion criteria 

The inclusion criteria encompassed all adult in-patients admitted to the University Hospital Lewisham (UHL) site for treatment within the orthopedics department during the period spanning from March 2022 to November 2022. The exclusion criteria, on the other hand, consisted of two primary categories. First, "Active Outliers" pertained to patients who exclusively received consultations from the orthopedics team without progressing to further treatment. Second, "Pediatric Patients" entailed the exclusion of individuals who fell below the age of adulthood from the analytical scope.

Data gathering

The first cycle of the audit was conducted in March 2022, with a sample size of 27 patients. Afterwards, interventions were made in the form of an institutional algorithm aimed at improving patient outcomes. The second cycle of the audit was conducted in November 2022, with a smaller sample size of 23 patients. Results were recorded using a standardized worksheet and analyzed using basic statistical methods.

**Figure 1:**
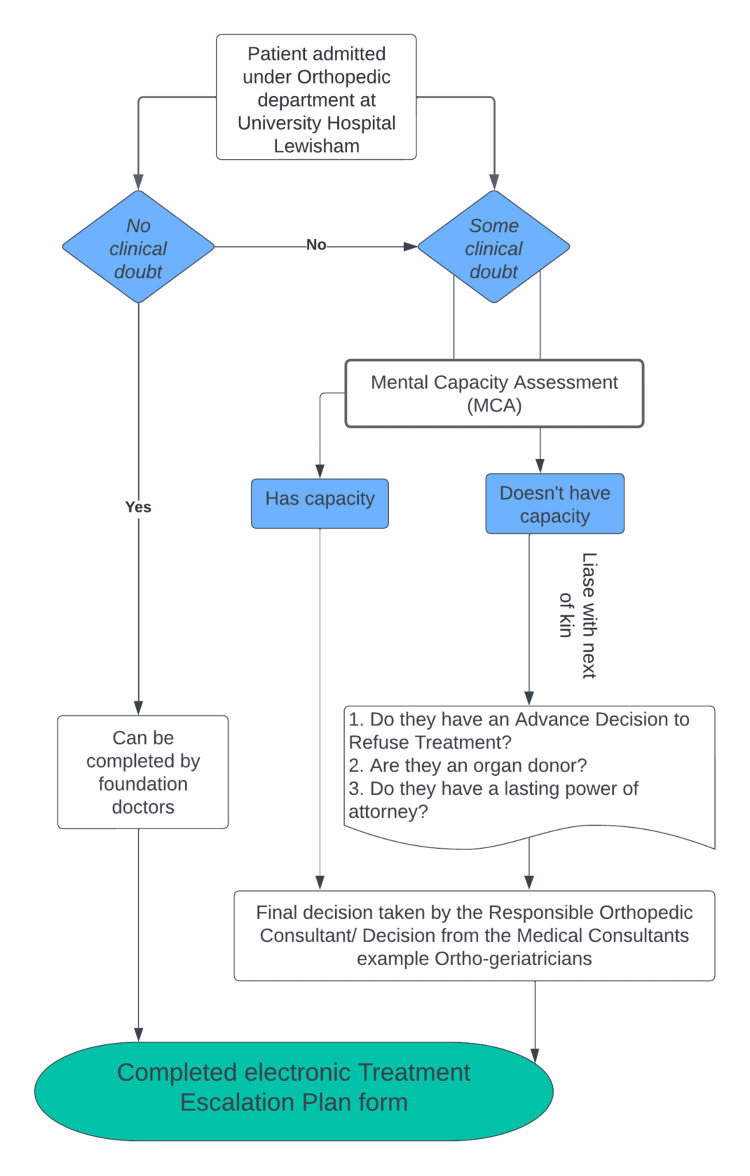
Local revised TEP pathway has been established for patients admitted to the orthopedic department at University Hospital Lewisham

The original protocol pertaining to the fulfillment of teaching evaluation processes (TEPs) stipulated that exclusively those trainees at the level of specialty trainees year 3 (ST3+) and beyond were eligible to undertake TEPs. Subsequent to the initial iteration of this protocol and subsequent deliberations during the routine governance assembly, a slight revision was introduced. This modification extended the privilege of engaging in TEP submissions to house officers (F1+) and higher designations, specifically in cases involving young patients afflicted with uncomplicated fractures (Figure 1). This modification was kept in line with the trust guidelines, where the final decision was discussed with a senior.

## Results

During Cycle 1, it was observed that only 47.82% of inpatients had their treatment escalation plan (TEP) completed within the specified timeframe of 48 hours, with an overall completion rate of 69.5%. The average duration of completing the TEP was found to be 4.4 days. Moreover, the average number of overrides recorded was 3.2 times, indicating instances where the TEP guidelines were bypassed. Notably, there was a particular case where a patient's TEP remained incomplete for a prolonged period of 35 days, with 24 instances of skipping the completion process (Table 1).

**Table 1:** TEP compliance in cycle 1 and 2 TEP: Treatment Escalation Plan

Parameter	Cycle 1	Cycle 2
Overall Completion Rate	69.5%	88.2%
Percentage Completed within 48 Hours	47.82%	35.29%
Average Time to Complete TEP (days)	4.4	3.7
Average Number of Overrides	3.2 times	1.3 times
Longest TEP Completion Duration (days)	35	13
Instances of Skipping Completion Process	24	5

In order to investigate the reasons behind the incomplete TEP forms, a questionnaire was administered to junior doctors. The purpose of this survey was to gather insights into the factors contributing to the non-completion of TEPs. It was found that healthcare professionals occasionally skipped the TEP form due to overriding clinical priorities. Among the completed inpatient TEPs, 50% were done by senior house officers (SHOs), 25% were done by registrars, 18.75% were completed by foundation year 1 (F1) doctors, and the remaining forms were completed by consultants.

Based on the findings, the team conveyed the message that young patients with simple fractures could have their TEPs completed at a junior level. This led to the team flagging incomplete TEPs to senior doctors for further attention and completion. Additionally, an algorithm was displayed in the trauma room, accompanied by email notifications sent to doctors outlining the algorithm's details. Individual teaching sessions were conducted to address specific concerns and challenges faced by colleagues. Furthermore, the team was reminded to discuss the TEP status with consultants during their post take ward round (PTWR).

Cycle 2 revealed that only 35.29% of inpatients had their TEP completed in accordance with the trust guidelines, while the overall completion rate reached 88.2%. The average time taken to complete the TEP was reduced to 3.7 days. The average number of overrides decreased to 1.3 times. The highest duration for completing a TEP was observed to be 13 days, with five instances of skipping the completion process (Table 1). 

## Discussion

The awareness and understanding of the approach to completing treatment escalation plan (TEP) forms among junior doctors have been found to have a notable impact on the rates of TEP completion. In order to ensure the continued effectiveness of the TEP completion process, it is crucial to implement regular auditing and training sessions for rotating junior doctors. These sessions will serve to reinforce their knowledge and understanding of the TEP guidelines, keep the process in check, and promote consistent and accurate completion of TEP forms.

The utilization of template for escalation of care (TEP) proformas has been embraced by various acute care facilities within the NHS with marked success. In a prior quality improvement initiative, these proformas exhibited the highest utilization rates in the context of acute care wards catering to elderly patients, thereby underscoring their significance in the context of patients characterized by increased frailty [5].

A separate project yielded noteworthy findings, revealing that 96% of the sampled patients and their respective family members expressed the view that the employment of TEP proformas would yield substantial benefits [2]. Furthermore, another project highlighted the insufficiency of do not attempt cardiopulmonary resuscitation (DNACPR) forms as a means of guiding clinical decisions pertaining to treatment escalation. In this context, it was evident that TEP proformas not only conferred advantages upon the patients but also proved beneficial to the attending clinicians, particularly junior doctors who were on-call [6].

In the contemporary landscape characterized by the ubiquity of electronic patient records, an argument can be made that reliance solely on paper based TEP proformas is antiquated. The implementation of TEP decisions within the framework of electronic patient records has been successfully demonstrated within the NHS, a development that finds support in our perspective [12].

The study yielded several critical lessons. First, there was a substantial improvement in the overall completion rate of treatment escalation plans (TEP) from 69.5% to 88.2% following the implementation of interventions. This improvement indicates the effectiveness of the strategies employed to address TEP completion issues. Second, the average time taken to complete TEPs notably decreased from 4.4 days to 3.7 days, demonstrating that the measures implemented successfully expedited the TEP completion process. Third, there was a reduction in the average number of overrides from 3.2 times to 1.3 times, reflecting the success of strategies in minimizing instances where TEP guidelines were bypassed.

Further research is needed to examine the impact of TEP completion rates on patient outcomes and healthcare costs, which can provide valuable insights into the clinical and economic benefits of completing TEP forms. Overall, the literature suggests that TEP completion rates are low in many healthcare settings, and interventions are needed to improve these rates. Healthcare providers may be more likely to complete TEP forms if they are given education and training on the process and provided with easy-to-use forms and clear instructions.

Several limitations of this study should be considered. The study was conducted at a single London teaching hospital, which may limit the generalizability of the findings to other healthcare settings. Additionally, the sample size was relatively small, with only 50 patients being included in the study. As a result, the study may have been underpowered to detect significant differences in TEP completion rates. It also did not examine the impact of TEP completion on patient outcomes or healthcare costs, which could have provided valuable insights into the clinical and economic benefits of completing TEP forms. The study also did not assess the long-term sustainability of the interventions implemented, but regular audits will keep TEP completion rates maintained over time.

## Conclusions

The study has demonstrated that interventions such as institutional algorithms and departmental meetings can be useful in improving the adherence of staff to complete TEPs. Ongoing training and education can help overcome some of the barriers to TEP completion.
